# Risk factors for mortality in culture-negative neonatal sepsis in Malawi: a propensity score-matched analysis

**DOI:** 10.1136/bmjpo-2024-002664

**Published:** 2024-06-21

**Authors:** Lughano Ghambi, James Chirombo, Tessa de Baat, Kondwani Kawaza, Pui-Ying Iroh Tam

**Affiliations:** 1 Malawi-Liverpool-Wellcome Trust Clinical Research Programme, Blantyre, Malawi; 2 Kamuzu University of Health Sciences, Blantyre, Southern Region, Malawi; 3 Liverpool School of Tropical Medicine, Liverpool, UK

**Keywords:** Child Health, Low and Middle Income Countries, Neonatology, Mortality

## Abstract

We conducted a propensity score-matched multivariable regression analysis of 1050 culture-negative neonatal sepsis cases in Malawi, where 160 (15.2%) died. Mortality among neonates with culture-negative sepsis was associated with very low birth weight (adjusted OR (AOR) 12.82, 95% CI 1.23 to 137.49), respiratory distress syndrome (AOR 13.20, 95% CI 2.58 to 83.66), a low Apgar score at 1 min (AOR 3.50, 95% CI 1.21 to 10.72) and at 5 min (AOR 4.77, 95% CI 1.94 to 12.50). Addressing maternal and perinatal factors around health and delivery of care is key to improving outcomes in the context of culture-negative sepsis in neonates from low-income country settings like Malawi.

Culture-negative neonatal sepsis in settings that lack diagnostic capacity contributes to excessive antimicrobial use and the rise of antimicrobial resistance (AMR),^1^ with the most rapid increase among young infants.^2^ Culture-negative neonatal sepsis in sub-Saharan Africa has been poorly described, due to the lack of quality-assured diagnostics as well as limited infrastructure. We aimed to describe the characteristics of neonates with culture-negative sepsis in Malawi and identify the risk factors associated with mortality.

We conducted a secondary analysis of blood and cerebrospinal (CSF) culture data collected between May 2018 and May 2019 in the neonatal unit at Queen Elizabeth Central Hospital in Blantyre, Malawi.^3^ All neonates who had cultures obtained with no growth of a significant pathogen were included in the analysis. We excluded those with a surgical condition (eg, hydrocephalus, myelomeningocele), necrotising enterocolitis or congenital malformations (eg, cardiac defects and syndromic). We collected microbiology and prescribing data, maternal and neonatal characteristics, and in-hospital mortality.

A propensity score with an exact matching method was performed using one-to-many matching to increase precision, to ensure the similarity of neonates in comparator groups, and to limit the bias of various prognostic factors. Propensity scores were estimated in a multivariable logistic regression model in R (V.4.3.1, 2023, Vienna, Austria: R Foundation for Statistical Computing). We included factors that were significant in the univariate analysis (known factors of neonatal mortality such as birth weight, gestation age, Apgar score, respiratory distress syndrome and admission temperature), as well as sex. A p<0.05 was considered statistically significant.

We had complete data for 1265 neonates out of 1406 who had a blood and/or CSF culture obtained (90.0%). Of those, 124 cultures were positive (9.8%), and 91 met the exclusion criteria (7.2%), leaving 1050 neonates in the unmatched cohort. There were deaths in 160 neonates (15.2%). On propensity score-matched analysis (table 1), neonates who died compared with those who survived were more likely to have low birth weight (51.6% vs 24.1%, p<0.01), prematurity (45.2% vs 17.4%, p<0.01), an Apgar score<7 (at 1 min (80.6% vs 46.2%), 5 min (50.0% vs 20.1%) and 10 min (45.9% vs 11.4%), p<0.01 for all), hypothermia (87.1% vs 54.7%, p<0.01), respiratory distress syndrome (38.7% vs 6.0%, p<0.01), birth asphyxia (45.2% vs 13.6%, p<0.01) and maternal syphilis (11.3% vs 3.8%, p=0.02). On multivariable logistic regression analysis (table 2), mortality in culture-negative neonatal sepsis was associated with very low birth weight (adjusted OR (AOR) 12.82, 95% CI 1.23 to 137.49, p=0.032), respiratory distress syndrome (AOR 13.20, 95% CI 2.58 to 83.66, p=0.003) and a low Apgar score at 1 min (AOR 3.50, 95% CI 1.21 to 10.72, p=0.023) and at 5 min (AOR 4.77, 95% CI 1.94 to 12.50, p=0.001).

**Table 1:** Characteristics of neonates with culture-negative sepsis that died versus survived

Characteristics	Unmatched cohort (N=1050)			Propensity score-matched (N=510)		
	Died (N=160)	Survived (N=890)	P value	Died (N=62)	Survived (N=448)	P value
Maternal characteristics						
Maternal age, median (IQR), years	23.00 (20.00–28.00)	23.00 (19.00–28.00)	0.85	22.50 (20.00–28.00)	23.00 (19.00–29.00)	0.54
Maternal HIV positive	27/149 (18.1%)	107/863 (12.4%)	0.07	7/62 (11.3%)	45/443 (10.2%)	0.82
Maternal antiretroviral therapy	24/25 (96.0%)	77/82 (93.9%)	1.00	6/6 (100.0%)	36/38 (94.7%)	1.00
Maternal syphilis positive	14/108 (13.0%)	32/648 (4.9%)	<0.01	7/62 (11.3%)	17/448 (3.8%)	0.02
Maternal receipt of benzathine penicillin	3/11 (27.3%)	5/20 (25.0%)	1.00	1/5 (20.0%)	4/9 (44.4%)	0.58
Maternal temperature ≥37.5°C	0/133 (0.0%)	12/133 (1.7%)	0.23	0/59 (0.0%)	7/406 (1.7%)	0.60
Maternal antimicrobials in labour	4/102 (3.9%)	63/501 (12.6%)	<0.01	3/46 (6.1%)	40/273 (12.8%)	0.24
Maternal antimicrobials in pregnancy	0/52 (0.0%)	7/549 (1.3%)	1.00	0/24 (0.0%)	3/261 (1.1%)	1.00
Intrapartum characteristics						
Mode of delivery						
Assisted	3 (1.9%)	24 (2.7%)	< 0.01	42 (67.7%)	251 (56.0%)	0.24
Caesarean section	31 (19.4%)	282 (31.7%)		2 (3.2%)	19 (4.2%)	
Spontaneous vaginal delivery	126 (78.8%)	584 (65.6%)		18 (29.0%)	178 (39.7%)	
Rupture of membranes >18 hours	9/87 (10.3%)	94/424 (22.2%)	0.01	5/39 (12.8%)	61/242 (25.2%)	0.11
Liquor						
Clear	94/117 (80.3%)	427/636 (67.1%)	<0.01	42/57 (73.7%)	243/381 (63.8%)	0.26
Meconium	20/117 (17.1%)	194/636 (30.5%)	<0.01	13/57 (22.8%)	126/381 (33.1%)	0.26
Offensive/bloody	3/117 (2.6%)	15/636 (2.4%)	<0.01	2/57 (3.5%)	12/381 (3.1%)	0.26
Neonatal characteristics						
Male sex	84 (52.5%)	489 (54.9%)	0.61	32 (51.6%)	244 (54.5%)	0.17
Gestational age <37 weeks	64/127 (50.4%)	168/777 (21.6%)	<0.01	28 (45.2%)	78 (17.4%)	<0.01
Apgar score <7						
At 1 min	101/134 (75.4%)	339/743 (45.6%)	<0.01	50 (80.6%)	207 (46.2%)	<0.01
At 5 min	61/133 (45.9%)	142/741 (19.2%)	<0.01	31 (50.0%)	90 (20.1%)	<0.01
At 10 min	30/70 (42.9%)	44/408 (10.8%)	<0.01	17/37 (45.9%)	30/263 (11.4%)	<0.01
Birth weight						
≥2500 g	57/158 (36.1%)	564/872 (64.7%)	< 0.01	30 (48.4%)	340 (75.9%)	< 0.01
1500–2499 g	41/158 (25.9%)	242/872 (27.8%)		16 (25.8%)	90 (20.1%)	
1000–1499 g	49/158 (31.0%)	64/872 (7.3%)		16 (25.8%)	18 (4.0%)	
<1000 g	11/158 (7.0%)	2/872 (0.2%)		NA	NA	
Admission temperature						
>37.5°C	7/152 (4.6%)	235/873 (26.9%)	<0.01	3 (4.8%)	113 (25.2%)	<0.01
<36.5°C	133/152 (87.5%)	443/873 (50.7%)	<0.01	54 (87.1%)	254 (54.7%)	<0.01
Intrauterine growth restriction	9/ (5.6%)	47 (5.3%)	0.85	4 (6.5%)	24 (5.4%)	0.76
Respiratory distress syndrome	78 (48.8%)	84 (9.4%)	<0.01	24 (38.7%)	27 (6.0%)	<0.01
Asphyxia	56 (35.0%)	104 (11.7%)	<0.01	28 (45.2%)	61 (13.6%)	<0.01
Continuous positive airway pressure	11 (6.9%)	24 (2.7%)	0.01	3 (4.8%)	7 (1.6%)	0.11
Kangaroo mother care	2 (1.2%)	19 (2.1%)	0.76	0 (0.0%)	11 (2.5%)	0.38
Neonatal antimicrobials duration, median (IQR), days	2.00 (1.00–3.00)	6.00 (5.00–7.00)	<0.01	2.00 (1.00–3.00)	6.00 (5.00–7.00)	<0.01

**Table 2:** Propensity score-matched multivariable logistic regression analysis of the association between neonatal culture-negative sepsis and mortality

Characteristic	Mortality	
	Propensity score matched	
	AOR	P value
Maternal syphilis positive	1.12	0.882
Maternal HIV positive	1.24	0.722
Assisted delivery	0.42	0.347
Caesarean delivery	0.64	0.295
Gestational age <37 weeks	0.58	0.577
Birth weight 1500–2499 g	1.91	0.426
Birth weight >1000–1499 g	12.82	0.032
Respiratory distress syndrome	13.20	0.003
Apgar score <7 at 1 min	3.50	0.023
Apgar score <7 at 5 min	4.77	0.001
Temperature >37.5°C	1.87	0.466
Temperature <36.5°C	2.92	0.068

AOR, adjusted OR.

In this propensity score-matched analysis of neonatal sepsis in tertiary hospitals in Malawi, we found that mortality in neonates with culture-negative sepsis was significantly associated with very low birth weight, respiratory distress syndrome and low Apgar scores. Therefore, optimising the provision of maternal and antenatal care is critical to improve early neonatal outcomes.^3^ Given the non-specific clinical presentation of acute illness in newborns, neonatal units witness antimicrobial usage that is 10 times higher in neonates with culture-negative sepsis than in those with proven infection.^4^ Overuse of antibiotics has been described as one of the major drivers for AMR.^1^ In high-income countries, prolonged antimicrobial exposure in culture-negative neonatal sepsis has been associated with adverse outcomes, including an increased risk of mortality.^5^ However, due to the large number of neonatal deaths in the first few days of life (86.9%), we were unable to evaluate this association in our dataset.

The study was limited by being from a single tertiary hospital, and therefore, findings may not be representative nor generalisable to the wider region. However, our use of propensity score matching would have limited the bias of other prognostic factors. Nonetheless, the strengths of this study are the large sample size, the routine and robust microbiological service, and the inclusion of 90% of patient records in the analysis. This study is, to our knowledge, the first comprehensive description and evaluation of risk factors for mortality in culture-negative neonatal sepsis in a low-income country in sub-Saharan Africa. Our findings highlight the importance of addressing maternal and perinatal factors around health and delivery of care in low-income country settings to improve neonatal outcomes.
